# Supplementary material for: ERG activates a stem-like proliferation-differentiation program in prostate epithelial cells with mixed basal-luminal identity
Source: bioRxiv. 2024 Apr 6:2023.05.15.540839. Originally published 2023 May 18. Preprint. [Version 2] doi: 10.1101/2023.05.15.540839 (PMC10996491; doi:10.1101/2023.05.15.540839)
Supplement: Supplement 1 [file NIHPP2023.05.15.540839v2-supplement-1.pdf]

bioRxiv preprint doi: https://doi.org/10.1101/2023.05.15.540839; this version posted April 6, 2024. The copyright holder for this preprint (which was not certified by peer review) is the author/funder, who has granted bioRxiv a license to display the preprint in perpetuity. It is made available under aCC-BY 4.0 International license.

Supplemental figures

bioRxiv preprint doi: https://doi.org/10.1101/2023.05.15.540839; this version posted April 6, 2024. The copyright holder for this preprint (which was not certified by peer review) is the author/funder, who has granted bioRxiv a license to display the preprint in perpetuity. It is made available under aCC-BY 4.0 International license.

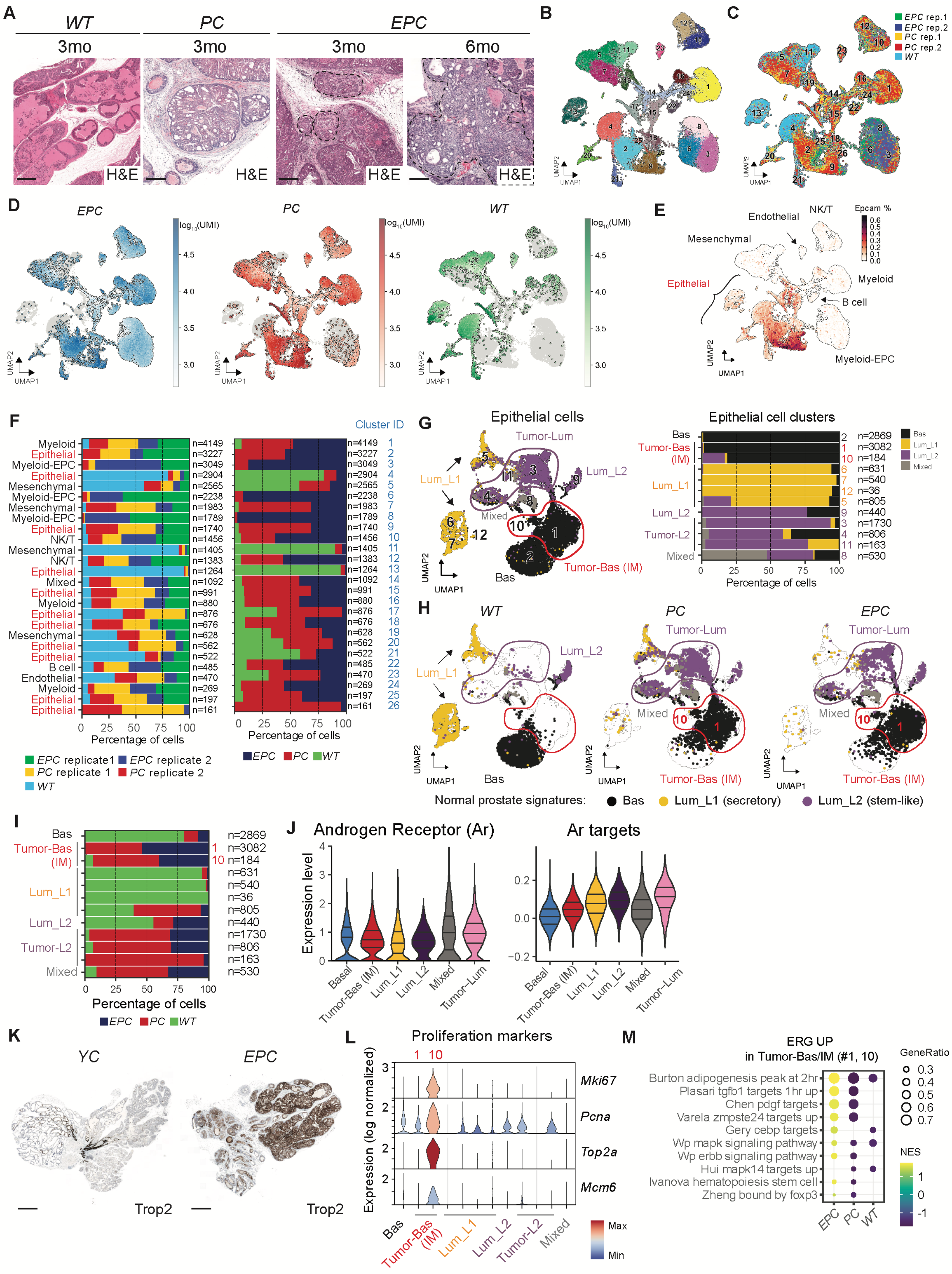

bioRxiv preprint doi: https://doi.org/10.1101/2023.05.15.540839; this version posted April 6, 2024. The copyright holder for this preprint (which was not certified by peer review) is the author/funder, who has granted bioRxiv a license to display the preprint in perpetuity. It is made available under aCC-BY 4.0 International license.

Figure S1. Time point characterization and annotation of scRNA-seq clusters of genetically engineered mouse models (GEMMs).

(**A**) H&E prostate histology. Dashed lines encircle invasive adenocarcinoma highlighting an ERG-dependent focal invasion from 3-month *EPC* mice that later became pervasive at 6 months age. Scale bars, 250 µm. (**B**) UMAP of all cells (N = 36,961 cells) from the prostates of 5 mice (2 *EPC*, 2 *PC*, 1 *WT*) annotated based on leiden clustering. (**C**) UMAP of all cells colored by individual replicates per genotype. (**D**) UMAP of all cells showing cells assigned to each genotype and colored by log10(UMI). **(E)** UMAP of all cells previously defined normal mouse prostate signatures. Epithelial cell clusters defined based on *Epcam* expression are highlighted. Myeloid cells specific to the *EPC* samples were defined as Myeloid-EPC. (**F**) Barplot of all cells colored by individual replicates (left) or each genotype with replicates aggregated (right). Numbers of cells in each cluster were included (n=) and cluster number corresponding to **B** is shown. (**G**) UMAP (left) and barplot (right) of all epithelial cells colored by cell types based on normal prostate signatures[35]. Numbers assigned to each individual cluster are shown. Colored circles highlight L2 cells (purple) and basal-like cells (red) specific to *PC* and *EPC* prostates (termed Tumor-Lum and Tumor-Bas respectively). Tumor-Bas cells were later defined as Tumor-IM cells as shown in **Fig. 1B**. A cluster containing a mix of different cell types is defined as mixed. Bas, basal; Lum, luminal. (**H**) UMAP showing epithelial clusters assigned to each genotype. Clusters are annotated as in **G**. The IDs of the two Tumor-Bas/IM clusters are shown. (**I**) Barplot of epithelial clusters showing the cell type composition of each cluster. Numbers of cells in each cluster were included. The clusters are numbered according to the UMAP in **G**. (**J**) Violin plots showing Androgen receptor (Ar) expression (left) and Ar signature scores (right) across epithelial cell types. (**K**) Sagittal view of whole prostates with Trop2 IHC on mice at 3 months age. In a normal prostate (*YC*), Trop2 selectively stains the stem-like L2 luminal cells at the proximal regions and distal invagination tips over the secretory L1 luminal cells, as expected[35,36,137,138]. By contrast, *EPC* tumor cells displayed a pan-Trop2 staining regardless of their tissue localization, further supporting a pervasive L2 transition as revealed from scRNA-seq analysis in **G-H**. Scale bars, 1 mm. (**L**) Violin plots comparing proliferative marker expression across all epithelial clusters. The two Tumor-Bas (IM) clusters are numbered according to the UMAP in **G**. (**M**) Gene set enrichment analysis showing EPC-specific pathways (ERG UP) within the Tumor-Bas/IM population.

bioRxiv preprint doi: https://doi.org/10.1101/2023.05.15.540839; this version posted April 6, 2024. The copyright holder for this preprint (which was not certified by peer review) is the author/funder, who has granted bioRxiv a license to display the preprint in perpetuity. It is made available under aCC-BY 4.0 International license.

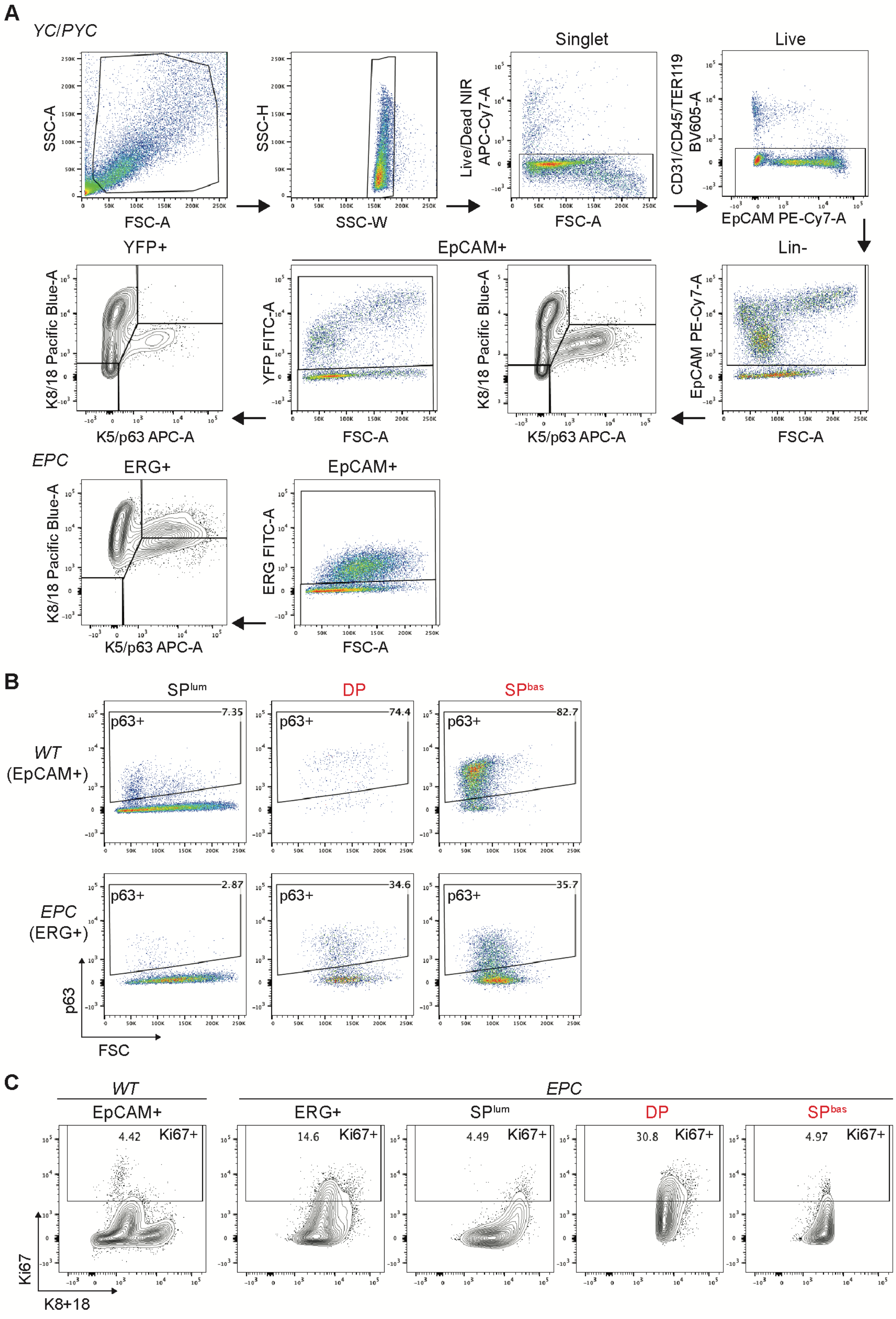

1 **Figure S2. Flow cytometry panel design.**

bioRxiv preprint doi: https://doi.org/10.1101/2023.05.15.540839; this version posted April 6, 2024. The copyright holder for this preprint (which was not certified by peer review) is the author/funder, who has granted bioRxiv a license to display the preprint in perpetuity. It is made available under aCC-BY 4.0 International license.

(**A**) Single cell suspension of dissociated prostate cells were gated for debris elimination, singlets (SSC-H vs -W), live cells (Live/Dead NIR exclusion), endothelial/immune exclusion (Lin-, CD31-,CD45-,Ter119-) and epithelial cells (EpCAM+). The epithelial population were further gated on the Cre-recombined population (YFP+ for *YC*/*PYC*, ERG+ for *EPC*) before analyzing basal (K5/p63 single positive, SP^{bas}), luminal (K8/18 single positive, SP^{lum}) and double-positive (DP) populations. DP population is gated as the outlier population based on the *WT* contour plot, assuming that DP cells rarely exist in normal adult prostates. The continuous population of SP^{bas} and DP in tumor samples (*PYC*/*EPC*) are combined and named as IM. (**B**) To assess p63 expression, epithelial or ERG/YFP+ cells were first gated as in **A**, before applying the p63+ gate to populations of interest. (**C**) To assess Ki67 expression, epithelial or ERG/YFP+ cells were first gated as in **A**. Ki67-positive cells were gated as the outlier population based on the *WT* contour plot, assuming that normal adult prostates are rarely proliferative, before applying to other populations of interest.

bioRxiv preprint doi: https://doi.org/10.1101/2023.05.15.540839; this version posted April 6, 2024. The copyright holder for this preprint (which was not certified by peer review) is the author/funder, who has granted bioRxiv a license to display the preprint in perpetuity. It is made available under aCC-BY 4.0 International license.

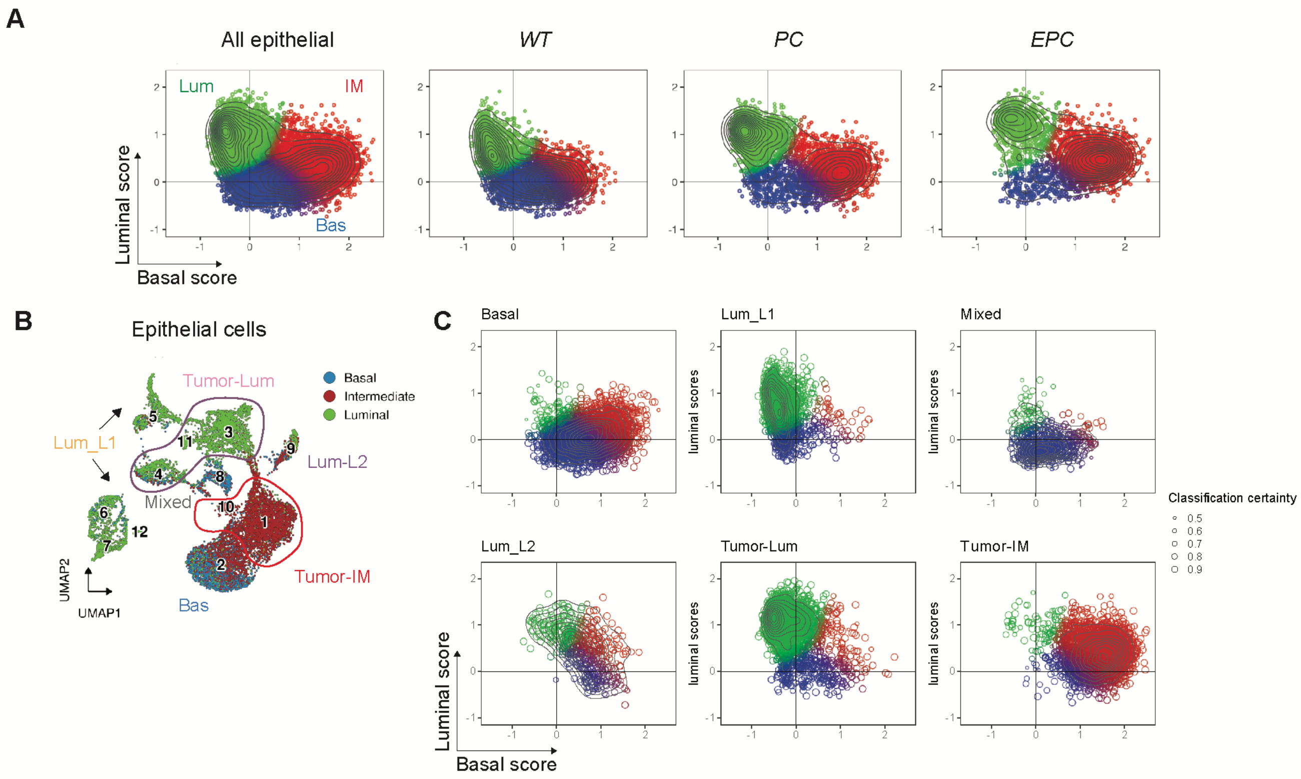

1 **Figure S3. Tumor-IM cells show intermediate features.**

2 (**A**) Assignment of basal and luminal scores to epithelial cells from all samples aggregated and each individual genotype (see methods). Unsupervised analysis revealed three distinct populations from all epithelial cells which were named as basal, luminal, and intermediate according to the marker scores. A pronounced double-positive IM population in *PC* and *EPC* mice are shown. (**B**) UMAP of all epithelial cells highlighting the IM identity of the Tumor-IM clusters. Clusters are colored by basal, luminal, and intermediate cell types defined in **A**. (**C**) Assignment of basal and luminal scores in each individual cell type.

bioRxiv preprint doi: https://doi.org/10.1101/2023.05.15.540839; this version posted April 6, 2024. The copyright holder for this preprint (which was not certified by peer review) is the author/funder, who has granted bioRxiv a license to display the preprint in perpetuity. It is made available under aCC-BY 4.0 International license.

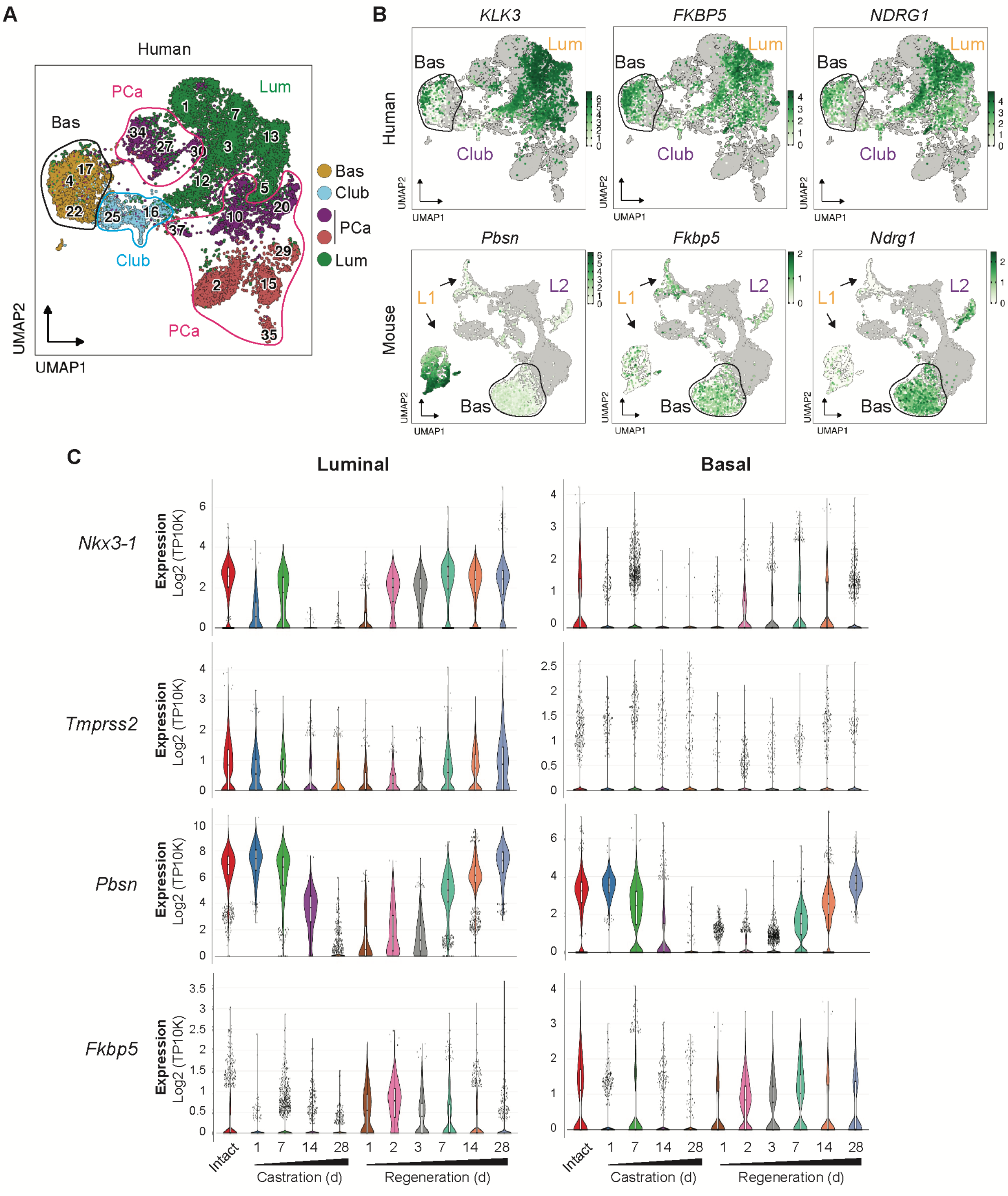

Figure S4. A subset of basal cells express androgen-regulated luminal genes.

(**A**) UMAP of all epithelial cells from human prostates. Data were reproduced from a previous study[54] with the same cell type classification, except that the two subtypes of the previously defined prostate cancer cells are together named as PCa here (see methods). The basal and PCa

bioRxiv preprint doi: https://doi.org/10.1101/2023.05.15.540839; this version posted April 6, 2024. The copyright holder for this preprint (which was not certified by peer review) is the author/funder, who has granted bioRxiv a license to display the preprint in perpetuity. It is made available under aCC-BY 4.0 International license.

clusters are highlighted in black and pink circles, respectively. (**B**) UMAP of epithelial cells from normal human and mouse prostates highlighting the expression of canonical luminal genes in a subset of basal cells. An expanded list of genes beyond **Fig. 2A** are shown. Normal basal clusters are highlighted in circles. Cells from normal samples are colored from white to green based on gene expression, with cells from tumor samples in grey in the background. The complete UMAPs and cell type annotations are shown in **A** (human) and **Fig. 1B** (mouse). (**C**) Changes in expression of luminal genes expressed in a subset of basal cells during a castration-regeneration cycle, indicative of androgen dependent expression, as seen in canonical luminal cells. Data were mined from a previous publication[35].

bioRxiv preprint doi: https://doi.org/10.1101/2023.05.15.540839; this version posted April 6, 2024. The copyright holder for this preprint (which was not certified by peer review) is the author/funder, who has granted bioRxiv a license to display the preprint in perpetuity. It is made available under aCC-BY 4.0 International license.

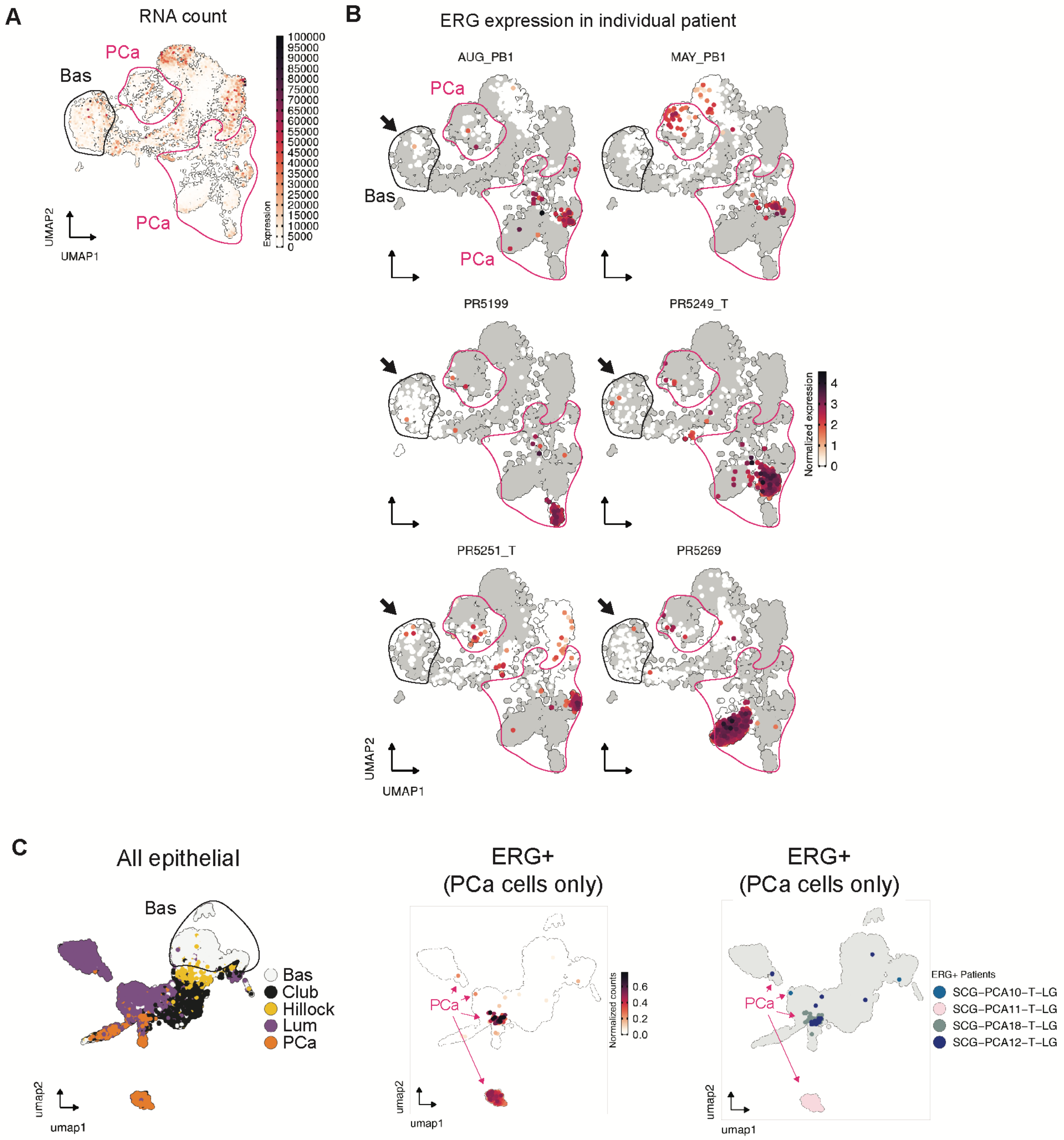

Figure S5. Evidence of ERG+ basal cells in human prostate cancer.
(**A**) UMAP of epithelial cells from patients[54] showing RNA count to assess doublet potential. The low to intermediate level of RNA count in basal cluster suggest the ERG+ basal cells in **Fig. 2E** are unlikely an artificial outcome of doublet formation. (**B**) UMAP of epithelial cells showing ERG expression in individual patients. ERG+ PCa samples are shown (see methods). Cells from each patient are colored from white to red based on gene expression, with cells from the rest of patients in grey in the background. Arrows highlight the presence of ERG+ cells in the basal cluster. Cell types in this figure are annotated based on **Fig. S4A**, with basal and PCa clusters highlighted in black and pink circles, respectively. (**C**) UMAP of epithelial cells from a different

bioRxiv preprint doi: https://doi.org/10.1101/2023.05.15.540839; this version posted April 6, 2024. The copyright holder for this preprint (which was not certified by peer review) is the author/funder, who has granted bioRxiv a license to display the preprint in perpetuity. It is made available under aCC-BY 4.0 International license.

patient cohort[53] showing all epithelial cells (left), ERG+ cancer cells colored by ERG expression (middle) and patient ID (right).

bioRxiv preprint doi: https://doi.org/10.1101/2023.05.15.540839; this version posted April 6, 2024. The copyright holder for this preprint (which was not certified by peer review) is the author/funder, who has granted bioRxiv a license to display the preprint in perpetuity. It is made available under aCC-BY 4.0 International license.

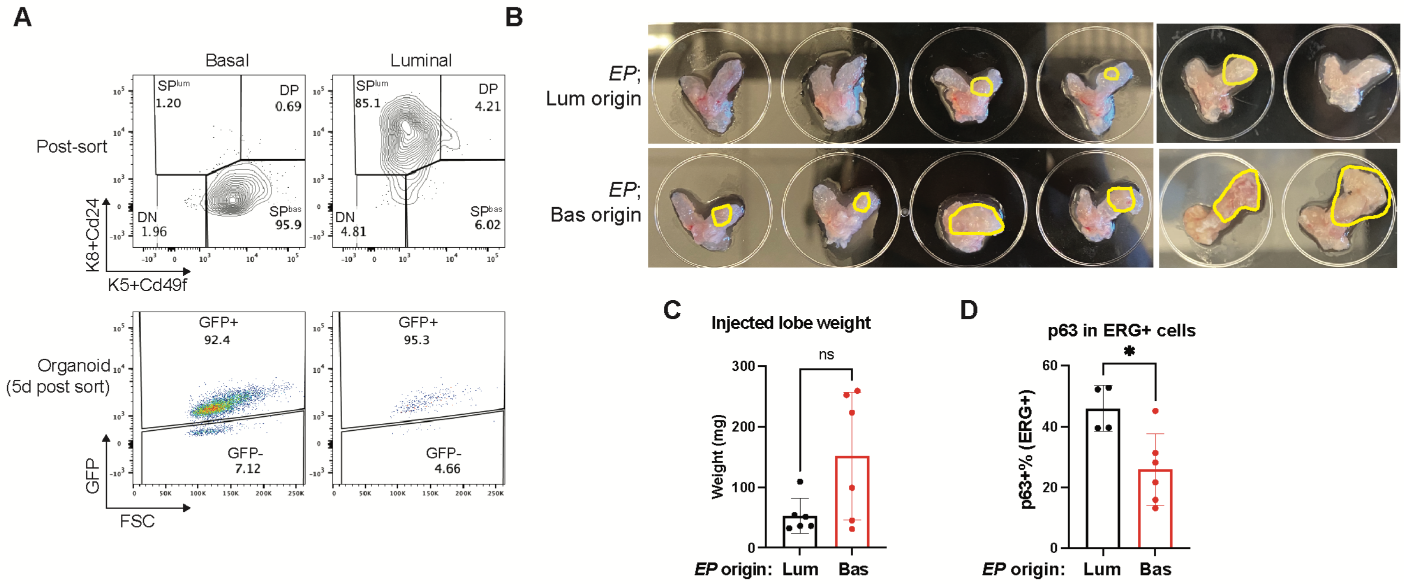

Figure S6. Generation and characterization of *EP* orthografts using freshly isolated and recombined cells.

(**A**) Quality control for freshly isolated basal and luminal-derived cells generated in **Fig. 3A**. (Top) Post-sort analysis to validate purity of the sorted basal and luminal populations. (Bottom) Assessment of Cre recombination efficiency using freshly derived organoids harvested at 5 days post Cre. GFP was used as a surrogate for ERG expression. (**B**) Images showing prostates harvested at 5 months post transplantation. Visible grafts, highlighted in yellow circles, suggest a higher graft burden from basal-derived *EP* orthografts. (**C**) Basal-derived *EP* orthografts trended towards a higher injected lobe weight than the luminal derivatives at the 5 months endpoint, further corroborating the higher graft burden as shown in **Fig. 3B**. (**D**) Basal-derived *EP* orthografts displayed a reduction of basal marker p63, further corroborating the notion of luminal fate transition as shown in **Fig. 3D**. p63 expression was measured by flow cytometry in ERG+ graft cells harvested at the 5 months endpoint. Data represent mean ± s.d.; n = 6 (except in **A** where n = 1); ns, not significant; *p<0.05; unpaired two-tailed t-test.

bioRxiv preprint doi: https://doi.org/10.1101/2023.05.15.540839; this version posted April 6, 2024. The copyright holder for this preprint (which was not certified by peer review) is the author/funder, who has granted bioRxiv a license to display the preprint in perpetuity. It is made available under aCC-BY 4.0 International license.

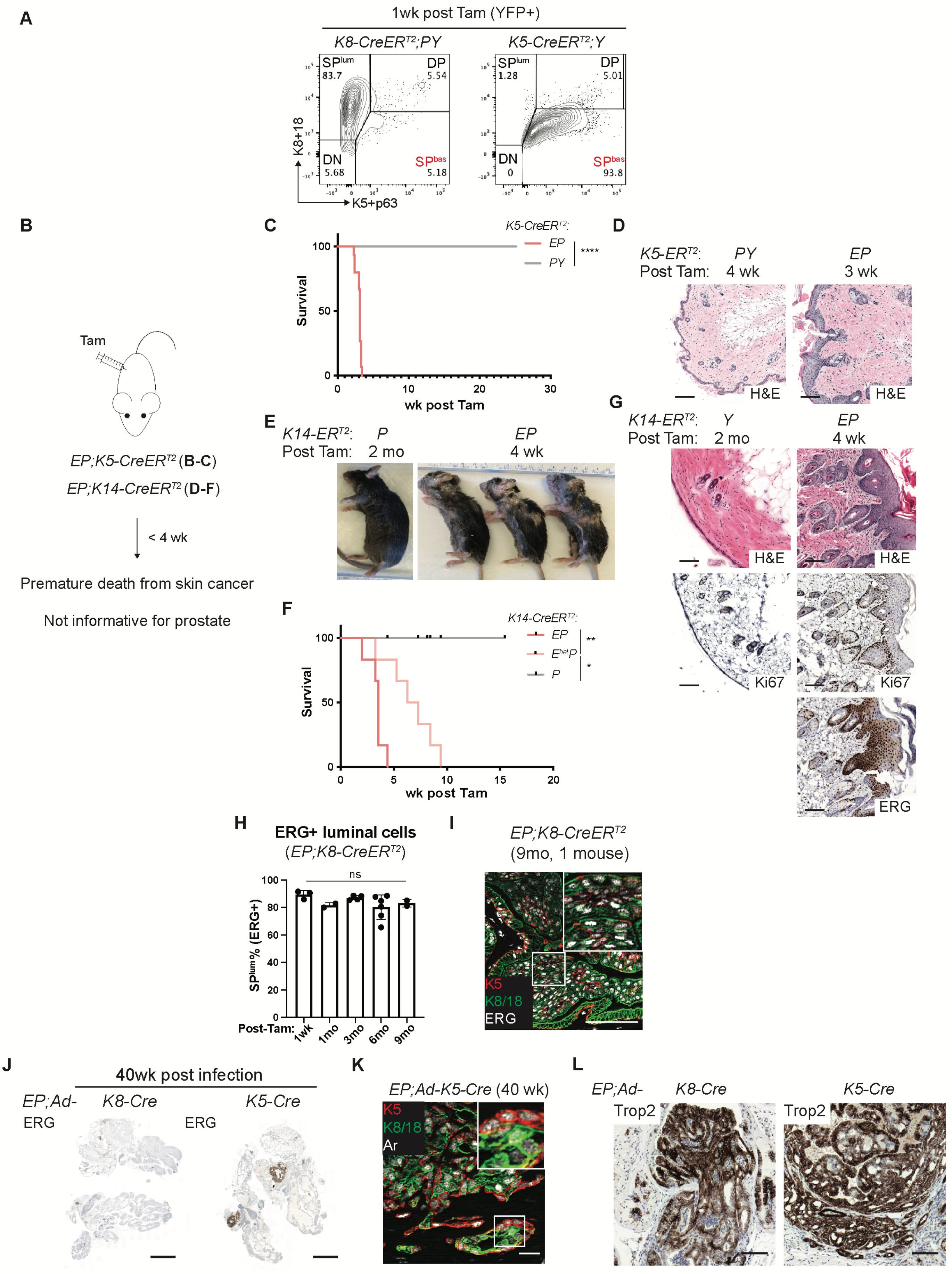

1

bioRxiv preprint doi: https://doi.org/10.1101/2023.05.15.540839; this version posted April 6, 2024. The copyright holder for this preprint (which was not certified by peer review) is the author/funder, who has granted bioRxiv a license to display the preprint in perpetuity. It is made available under aCC-BY 4.0 International license.

Figure S7. Lineage tracing comparing EP activation from basal and luminal cells.

(**A**) Characterization of the cell type specificity of *K5*- and *K8-CreER*^{T2}. The basal (SP^{bas}) and luminal (SP^{lum}) cells identity of the YFP+ recombined cells was measured by flow cytometry at 1 week post tamoxifen administration. (**B**) Schematic summarizing that lineage tracing using *K5* and *K14-CreER*^{T2} to activate *EP* leads to a severe skin disease and premature death. This precluded scoring any phenotype in the prostate. (**C-D**) Survival curve (**C**) and skin histology (**D**) using *K5-CreER*^{T2} highlighting an ERG-dependent skin cancer which led to early mouse death. Scale bar, 100µm. (**E-G**) Mice at harvest (**E**), survival curve (**F**) and skin histology (**G**) using *K14-CreER*^{T2} highlighting an ERG-dependent skin cancer which led to early mouse death, similar to the *K5-CreER*^{T2} model in **C-D**. The severity of the phenotype also correlated with ERG dosage. Ki67 IHC is shown to access the cell proliferation activity. Scale bar, 100µm. (**H**) Flow cytometry showing that ERG+ cells maintain luminal identity in *EP;K8-CreER*^{T2} mice through 9 months of tracing. (**I**) IF image showing rare foci of K5+ IM cells from one mouse at the 9 months endpoint. These cells were not clearly identified as a distinct population from flow cytometry in **Fig. 3F**, consistent with the rarity of these cells. Inset shows high-power view. Scale bar, 100µm. (**J**) Sagittal view of whole prostates with ERG IHC highlighting a marked increase in tumor volume in *EP* mice upon Ad-K5-Cre treatment relative to Ad-K8-Cre. Two injection sites are shown. Scale bar, 2mm. (**K**) IF showing Ar expression in both K5- and K5+ cells from invasive adenocarcinomas of *EP;Ad-K5-Cre* mice; inset shows high-power view. Scale bars, 20 µm. (**L**) Trop2 IHC showing a strong Trop2 positivity of the ERG+ lesions, suggesting that the tumor luminal cells acquire a L2 identity[35,36,138]. Trop2 was only detected in the invagination tips in the neighboring glands from adjacent sections from the same mice as in **Fig. 3K**. Note that Trop2 expression was also seen in *EP;Ad-K8-Cre* injected mice, indicative of *EP* activation, but in cells that are not capable of progressing to invasive adenocarcinoma. Scale bar, 100µm.

bioRxiv preprint doi: https://doi.org/10.1101/2023.05.15.540839; this version posted April 6, 2024. The copyright holder for this preprint (which was not certified by peer review) is the author/funder, who has granted bioRxiv a license to display the preprint in perpetuity. It is made available under aCC-BY 4.0 International license.

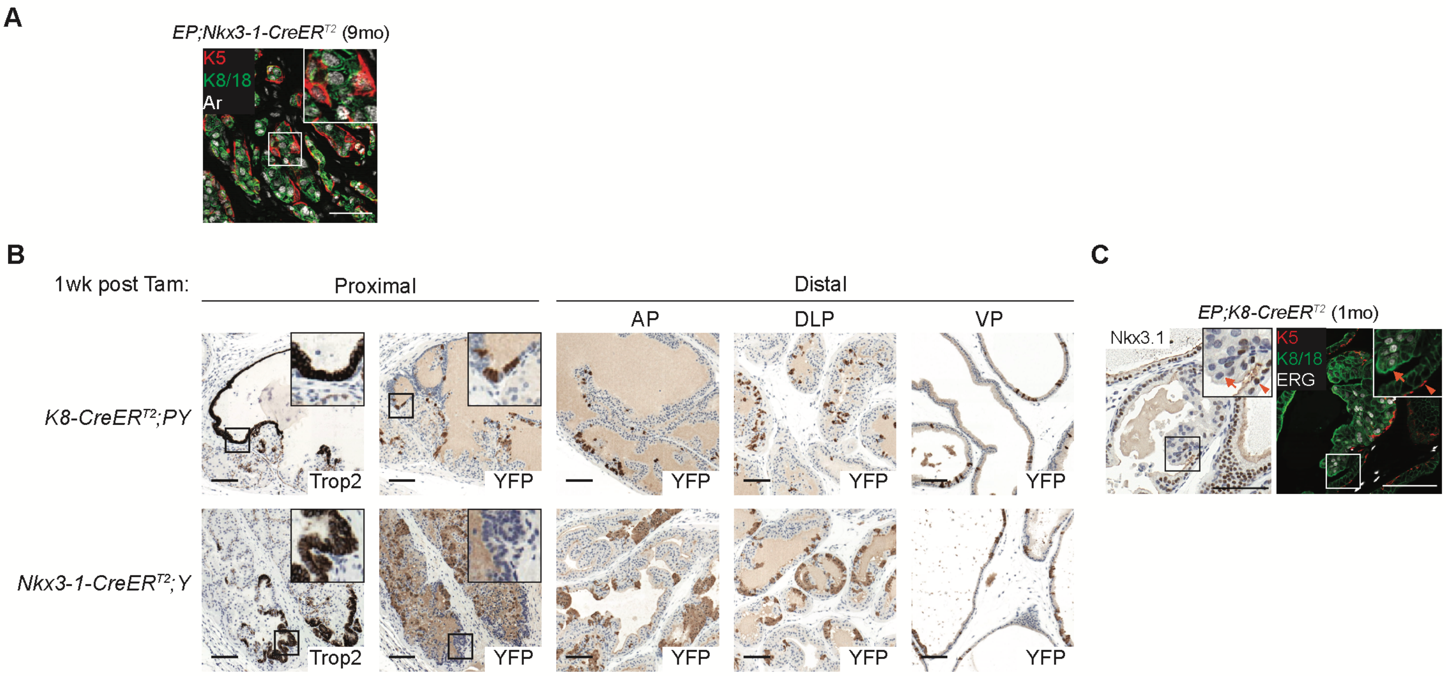

Figure S8. In situ analysis of lineage marker expression and Cre recombination efficiency
(**A**) IF staining showing Ar expression in both K5- and K5+ cells from invasive adenocarcinomas of indicated mice; inset shows high-power view. Scale bars, 50 µm. (**B**) IHC documenting recombined cells after crossing with *K8-CreER^{T2}* vs *Nkx3-1-CreER^{T2}*mice, YFP was used as a surrogate for Cre recombination. Scale bars, 100 µm. (**C**) *EP;K8-CreER^{T2}* mice displayed Nkx3.1 loss in ERG+ cells from the precursor lesions by 1 month post tamoxifen. High-power view in insets highlights the absence of Nkx3.1 signal in ERG+ cells (arrow) and the neighboring Nkx3.1+ normal luminal cells. The *Nkx3-1-CreER^{T2}* allele causes haploinsufficiency of the tumor suppressor gene *Nkx3-1*[139] which could potentially explain the tumorigenic phenotypes in *EP;Nkx3-1-CreER^{T2}* mice in **Fig. 4**. However, the data here show an early loss of Nkx3.1 in *EP;K8-CreER^{T2}* mice as well (potentially reflecting an L2 transition and thus loss of the L1 marker Nkx3.1). Thus, *Nkx3-1* expression is similarly disrupted in both settings and therefore unlikely to cause the different phenotypes. Scale bars, 100 µm.

bioRxiv preprint doi: https://doi.org/10.1101/2023.05.15.540839; this version posted April 6, 2024. The copyright holder for this preprint (which was not certified by peer review) is the author/funder, who has granted bioRxiv a license to display the preprint in perpetuity. It is made available under aCC-BY 4.0 International license.

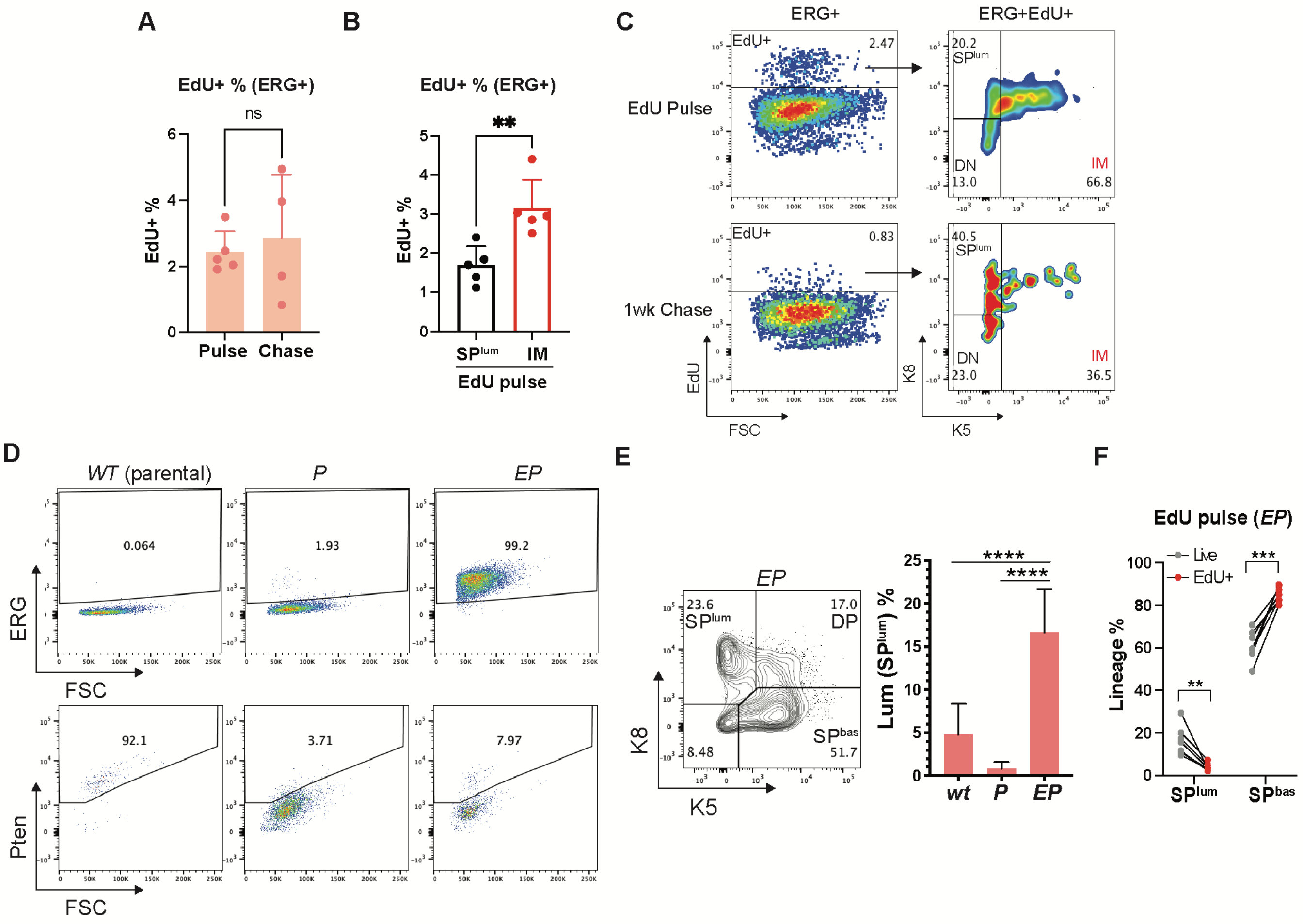

Figure S9. ERG+ basal and intermediate cells proliferate towards a luminal fate *in vitro* and *in vivo*.

(**A**) *In vivo* EdU pulse chase assay showing EdU quantification in ERG+ *EPC* cells. The percent of EdU-labeled cells were comparable between the pulse and chase samples, suggesting that the EdU+ population is not likely to enrich the label-retaining cells within 1 week of chase. (**B**) The same samples from **A** showing EdU quantification in SP^{lum} and IM populations of ERG+ *EPC* cells in pulse samples. IM cells showed a higher EdU signal, further corroborating the more proliferative feature of these cells as revealed in **Fig. 1**. (**C**) Flow cytometry analysis on ERG+ cells from **A** highlighting a SP^{lum} shift in EdU+ cells after 1 week of chase. (**D**) Flow cytometry validating the expected ERG and Pten expression status in *EP* organoids and the isogenic controls. (**E**) Flow cytometry showing an ERG-dependent expansion of luminal cells (SP^{lum}) in *EP* organoids. (**F**) Flow cytometry quantification in *EP* cells after a EdU pulse. The EdU+ population showed a depletion of luminal (SP^{lum}) cells and an enrichment of basal (SP^{bas}) cells relative to the total live population. Data represent mean ± s.d.; n > 3.; ns, not significant, *$p < 0.05$; **$p < 0.01$; ***$p<0.001$; ****$p < 0.0001$; unpaired two-tailed t-test (**A, B, E**); multiple paired t-test with FDR correction by Benjamini, Krieger and Yekutieli (**F**).

bioRxiv preprint doi: https://doi.org/10.1101/2023.05.15.540839; this version posted April 6, 2024. The copyright holder for this preprint (which was not certified by peer review) is the author/funder, who has granted bioRxiv a license to display the preprint in perpetuity. It is made available under aCC-BY 4.0 International license.

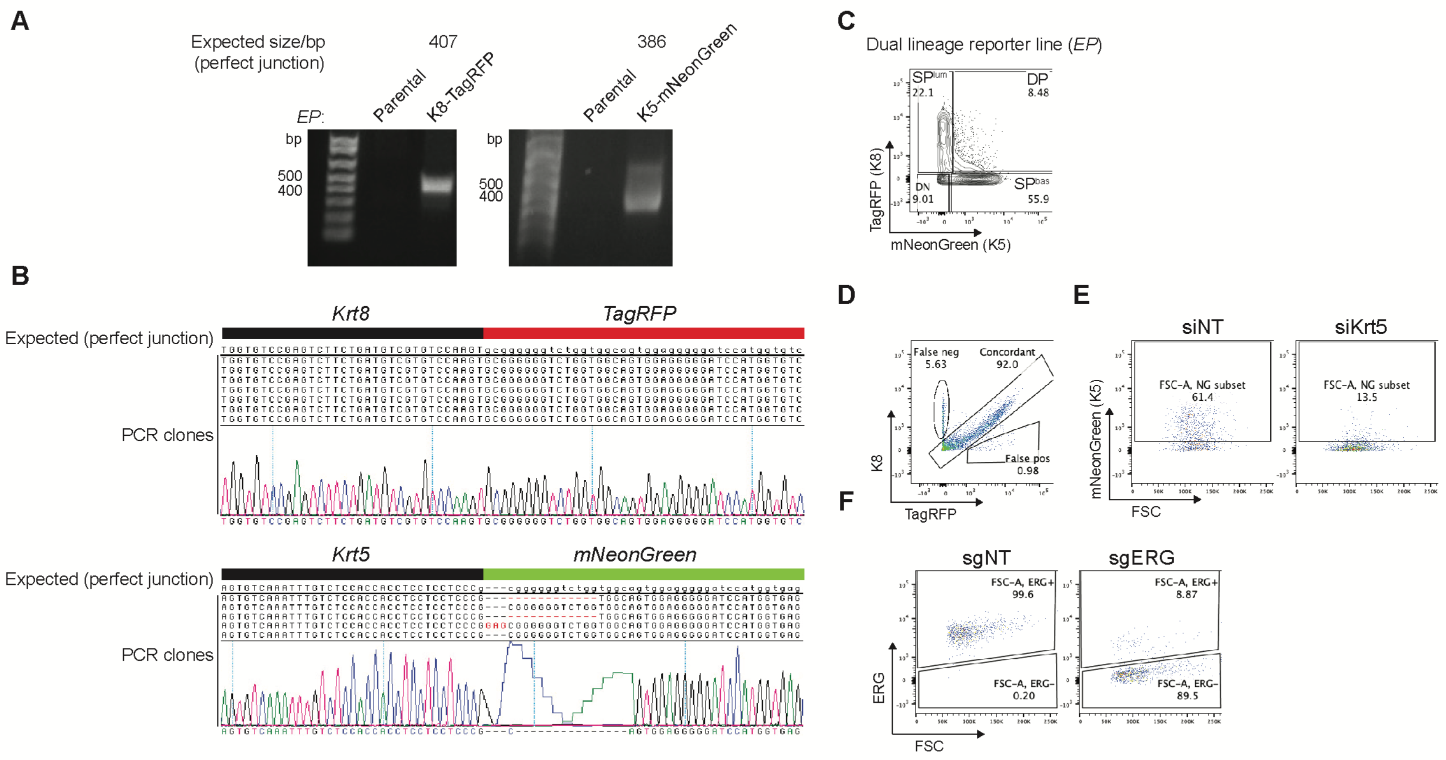

Figure S10. Generation and characterization of the dual lineage reporter knock-in system in *EP* organoids.

(**A**) Junction PCR across the target loci and the engineered reporters in the bulk organoid population. The PCR products specific to the knock-in allele with the expected size indicate successful reporter targeting. (**B**) Sanger sequencing of the junction PCR clones from **A** validates expected junction sequences with homogeneous perfect junctions for *Krt8-TagRFP* targeting, and heterogenous junctions with small in-frame insertion/deletions for *Krt5-mNeonGreen* targeting. (**C**) Live cell flow cytometry using the engineered reporter signals recapitulated a similar basal/luminal lineage pattern created by K5/K8 intracellular flow in **Fig. S9E**. (**D**) Intracellular flow cytometry comparing the expression pattern between the endogenous K8 and the TagRFP reporter signal in targeted organoid population. ~92% of overall concordance was observed. ~5.6% false-negative population was also observed which either reflects a lack of targeting or a disruption of protein function in a minority of cells. A similar assay was not possible with the *Krt5-mNeonGreen* targeting due to the epitope ablation by the targeting event. (**E**) Functional validation of the *Krt5-mNeonGreen* engineering by Krt5 depletion, which led to a reduction of the mNeonGreen reporter signal. (**F**) Effective ERG ablation by CRISPR in *EP* reporter organoids. ERG was detected by intracellular flow cytometry 2 days after introducing the indicated CRISPR-RNP.

bioRxiv preprint doi: https://doi.org/10.1101/2023.05.15.540839; this version posted April 6, 2024. The copyright holder for this preprint (which was not certified by peer review) is the author/funder, who has granted bioRxiv a license to display the preprint in perpetuity. It is made available under aCC-BY 4.0 International license.

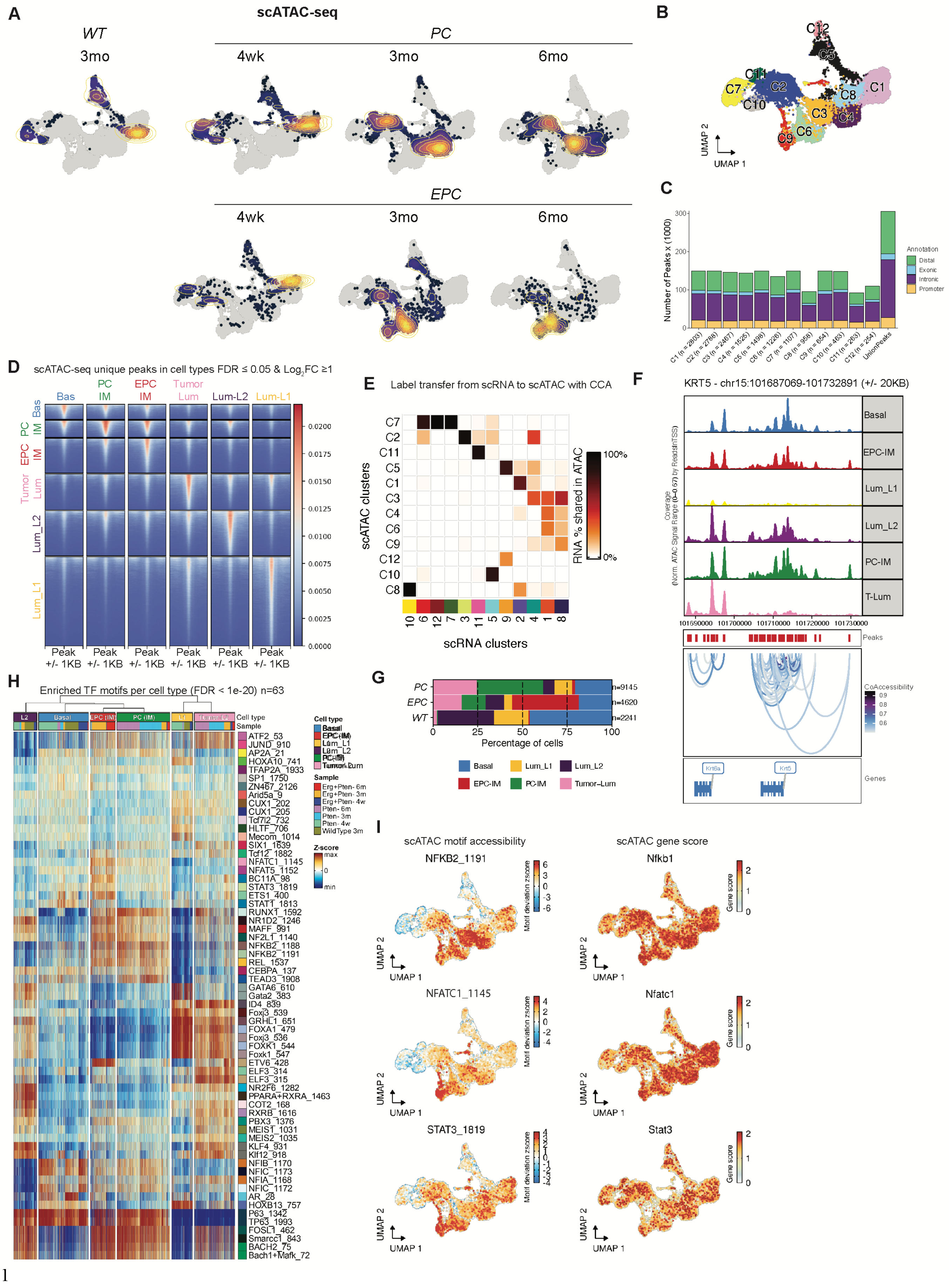

bioRxiv preprint doi: https://doi.org/10.1101/2023.05.15.540839; this version posted April 6, 2024. The copyright holder for this preprint (which was not certified by peer review) is the author/funder, who has granted bioRxiv a license to display the preprint in perpetuity. It is made available under aCC-BY 4.0 International license.

Figure S11. ERG drives a unique chromatin state in intermediate cells.

(A) scATAC-seq UMAPS by indicated genotypes and time points. Cell density is overlaid and colored by increasing density, from dark to light. **(B)** Clustering generated by leiden algorithm is shown labeled and also colored on scATAC-seq UMAP. **(C)** Barplot showing number of peaks annotated as distal, exonic, intronic, or promoter by cluster. **(D)** Tornado plot showing significantly enriched peaks (FDR <= 0.05 and Log2FC >= 1) for each annotated cell type (Bas, PC-IM, EPC-IM, Tumor-Lum, Lum_L2, Lum_L1) vs all other cell types. Signal plotted is normalized scATAC-seq aggregated by cell type, centered at peak and extended by 1000 nt up and down. **(E)** Grid showing amount of RNA shared with scATAC-seq by cluster. Labels from scRNA were transferred to scATAC using Seurat's CCA. **(F)** scATAC-seq reads piled up per cell type around the mm10 *KRT5* locus, a classical basal cell marker (top panel). As expected, signal in Lum_L1 is low compared to Basal. Second panel from top shows tick marks to indicate detected peaks. Third panel from top show inferred Co-Accessibility using Cicero, an algorithm used to predict DNA interactions from scATAC-seq data. Fourth panel from top shows *Krt5* gene locus and neighboring *Krt6a* locus in mouse. **(G)** Barplot percentage of cell type per genotype is shown. **(H)** scATAC-seq heatmap of most enriched transcription factor candidates when comparing one cell type to all others. Cells are grouped by cell type as columns and rows indicate motifs from database. The heatmap elements are colored by chromvar inferred z-score. Motif significance was calculated (see methods) and those having an p-value < 1e-20 after BH-false discovery rate correction are shown. **(I)** Representative UMAPs colored by motif deviation z-score (left) and inferred gene expression (gene score, right) of EPC-IM enriched TFs (NFKB2, NFATC1, STAT3).
